# Disruption of the Nitric Oxide Reductase Operon via *norD* Deletion Does Not Affect *Brucella abortus* 2308W Virulence

**DOI:** 10.3390/microorganisms13122875

**Published:** 2025-12-18

**Authors:** Faisal Rasheed, Amaia Zúñiga-Ripa, Miriam Salvador-Bescós, Hamid Irshad, Raquel Peña-Villafruela, Pilar M. Muñoz, María Jesús de Miguel, Qurban Ali, Raquel Conde-Álvarez, Saeed-ul-Hassan Khan

**Affiliations:** 1 Department of Zoology, Faculty of Biological Sciences, Quaid-i-Azam University Islamabad, Islamabad 44000, Pakistan; faisalqaisrani77@gmail.com; 2 Department of Microbiology and Parasitology, Instituto de Investigación Sanitaria de Navarra (IdiSNA), University of Navarra, 31008 Pamplona, Spain; azuniga@unav.es (A.Z.-R.); msalvadorb@unav.es (M.S.-B.); rpenavillaf@alumni.unav.es (R.P.-V.); 3 National Reference Laboratory for Poultry Diseases, Animal Sciences Institute, National Agricultural Research Centre, Islamabad 44000, Pakistan; hamidirshad@hotmail.com; 4 Departamento de Ciencia Animal, Centro de Investigación y Tecnología Agroalimentaria de Aragón (CITA), 50013 Zaragoza, Spain; pmmunnoz@cita-aragon.es (P.M.M.); mjmiguel@cita-aragon.es (M.J.d.M.); 5 National Veterinary Laboratories, Park Road, Islamabad 45710, Pakistan; drqurban@yahoo.com

**Keywords:** *Brucella*, *nor* operon, denitrification, nitrosative stress, nitrate respiration

## Abstract

*Brucella* are intracellular pathogens that use flexible respiratory strategies to adapt to oxygen-limited conditions. The *nor* operon encodes components of nitric oxide reductase (Nor), which are involved in denitrification and nitric oxide (NO) detoxification. In this study, the role of the *norD* gene in nitrate-dependent respiration, resistance to nitrosative stress, and intracellular persistence in *B. abortus* was evaluated. A non-polar Δ*norD* mutant was generated in strain 2308W and its survival and growth under aerobic and anaerobic conditions, with and without nitrate, as well as its tolerance to NO donors, were analyzed. In addition, its behavior was evaluated in activated and non-activated murine RAW264.7 and human THP-1 macrophages and in a murine infection model. The deletion of *norD* did not affect viability or growth under any of the conditions tested, nor did it alter resistance to NO in vitro or within activated macrophages. Furthermore, the mutant showed virulence comparable to the wild-type strain in BALB/c mice. These results contrast with those described for other *Brucella* species, suggesting that *norD* is dispensable in *B. abortus* 2308W virulence and that in the *Brucella* genus, there are species-specific differences in the role of the *nor* operon during infection.

## 1. Introduction

Brucellosis is a globally distributed zoonosis caused by Gram-negative bacteria of the genus *Brucella*, posing a significant threat to both animal health and human welfare [1,2]. The core brucellae include species infecting terrestrial and marine mammals (*B. abortus*, *B. melitensis*, *B. suis*, *B. canis*, *B. ovis*, *B. neotomae*, *B. microti*, *B. ceti*, and *B. pinnipedialis*), whereas the non-core group comprises genetically divergent strains isolated from amphibians, fish, and other non-mammalian or environmental sources [3,4,5]. Among the core classical species, *B. melitensis*, *B. abortus*, and *B. suis* are the most relevant zoonotic agents, responsible for the majority of human and livestock infections worldwide [6,7].

*Brucella* spp. are facultative intracellular pathogens capable of invading and replicating within both professional phagocytes and non-phagocytic host cells. Their virulence relies on the ability to evade early innate immune recognition and to establish a replicative niche within an endoplasmic reticulum-derived vacuole, a process mediated by a type IV secretion system that delivers effector proteins into the host cytosol. Within this specialized compartment, *Brucella* multiplies extensively, reflecting a high degree of metabolic adaptation to the nutrient- and oxygen-limited intracellular environment [8,9,10,11,12,13].

**Figure 1:**
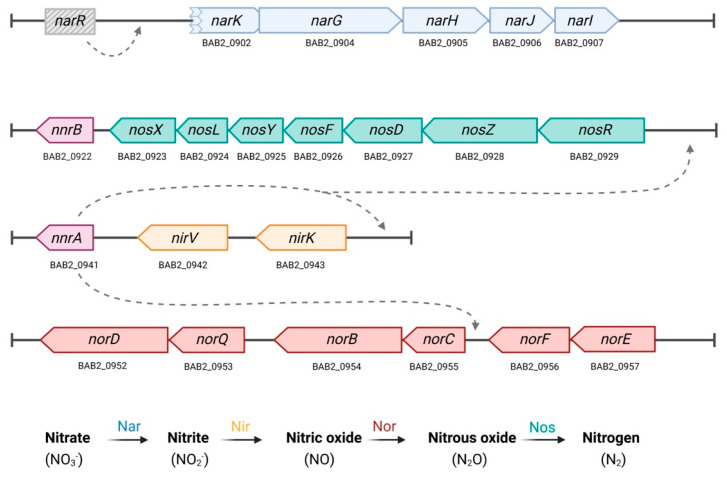
Schematic representation of the *Brucella abortus* 2308 denitrification gene clusters. The *nar*, *nir*, *nor*, and *nos* operons putatively encode enzymes responsible for the sequential reduction of nitrate (NO_3_^−^) to dinitrogen (N_2_) through nitrite (NO_2_^−^), nitric oxide (NO), and nitrous oxide (N_2_O). Genes are grouped and color-coded according to their function in the denitrification pathway: *nar* (light blue), *nir* (orange), *nor* (red), and *nos* (green). Regulatory genes *nnrA* and *nnrB* are shown in purple, while *narR* (gray) is absent in *B. abortus* 2308. Dashed arrows indicate proposed regulatory interactions. Adapted from Haine et al. 2006 [26].

Successful replication under these restrictive conditions may require flexible energy-generating strategies that enable the bacterium to adapt to fluctuating oxygen levels. Such respiratory flexibility enables the use of alternative electron acceptors, such as nitrogen oxides or sulfate, facilitating survival under microaerobic or anaerobic conditions. Although *Brucella* species have long been considered strict aerobes [14,15], nitrate–nitrite reduction has been reported in all classical species except *B. ovis* [16,17,18]. Based on this, it was hypothesized that the brucellae encounter a microaerobic environment within the intracellular vacuoles and may respire nitrate to sustain survival and growth [19]. Supporting this view, Freddi et al. have recently demonstrated that some members of the genus can grow under anaerobic conditions by using nitrate (NO_3_^−^) as an alternative electron acceptor via a denitrification pathway [20]. This pathway involves four operons, *nar*, *nir*, *nor*, and *nos*, that sequentially reduce nitrate (NO_3_^−^) to dinitrogen gas (N_2_). The *nar* operon encodes nitrate reductase (Nar), which converts nitrate into nitrite (NO_2_^−^); *nir* operon encodes nitrite reductase (Nir), which reduces NO_2_^−^ to nitric oxide (NO); *nor* encodes nitric oxide reductase (Nor), which converts NO to nitrous oxide (N_2_O); and finally, *nos* encodes nitrous oxide reductase (Nos), which completes the pathway by reducing N_2_O to N_2_ (Figure 1). These authors showed that the atypical core species *B. microti* exhibits robust anaerobic growth in nitrate-supplemented media with rapid nitrite turnover, indicative of efficient denitrification, while *B. suis* displays limited anaerobic growth and nitrite accumulation, suggesting a slower or incomplete denitrification process [20]. In contrast, the classical species *B. abortus* and *B. melitensis* showed only marginal anaerobic proliferation, consistent with reduced denitrifying capacity. These findings point to functional diversity within the core clade regarding respiratory flexibility and nitrate metabolism.

Beyond its role in energy generation under low-oxygen conditions, the *nor* operon might also contribute to the detoxification of nitric oxide (NO), a key component of the nitrosative burst generated by activated macrophages [21]. NO and other reactive nitrogen species (RNS) can damage bacterial DNA, proteins, and membranes, and the Nor complex plays a crucial role in counteracting these nitrosative stress, promoting intracellular survival by limiting NO accumulation and mitigating immune-mediated damage.

In this study, we investigated the role of the *norD* gene, a component of the *nor* operon, in *B. abortus* 2308W with respect to nitrate-dependent respiration, resistance to nitrosative stress, and intracellular persistence. Based on biochemical studies in *Paracoccus denitrificans*, NorD is an accessory factor whose precise role remained unclear for a long time, but is now understood to function together with NorQ to facilitate insertion of the non-heme iron FeB cofactor into NorB, a step that is essential for nitric oxide reductase activity [22]. Early biochemical studies in *B. abortus* strain 19 by Rest and Robertson, and Sperry and Robertson demonstrated the presence of a membrane-linked nitrate reductase capable of supporting metabolism under oxygen-limiting conditions, while nitrite alone had no stimulatory effect [23,24]. These findings suggested that *B. abortus* may rely on nitrate reduction without functional downstream pathways for nitrite respiration. Nevertheless, this does not preclude the possibility that the *nor* operon, and specifically NorD, contributes to nitric oxide detoxification. In this sense, studies in other *Brucella* species support a clear role for the *nor* operon in detoxification of host-derived NO. In *B. suis* 1330, a ΔnorD mutant is attenuated in activated macrophages and in mice, but not in resting cells [25], and similarly, in *B. melitensis* 16M, the deletion of *norB* compromises survival in activated macrophages and reduces persistence in vivo [26]. However, the contribution of the *nor* genes to *B. abortus* physiology and virulence remains understood and the physiological role of the *nor* genes in this species has not been defined. Despite this, *norD* has already been exploited, together with *znuA* deletion, in a *B. abortus* 2308 background to generate a protective vaccine candidate that conferred protection against *B. abortus* challenge [27,28].

Since the physiological role of NorD in *B. abortus* remains unexplored, our objective was to define its contribution to bacterial metabolism and persistence. To this end, we generated a ΔnorD mutant and evaluated its fitness under different oxygen and nitrate conditions, its survival within activated and non-activated macrophages, and its virulence in a murine model. By integrating these complementary approaches, we sought to determine whether B. abortus depends on *nor* genes in a manner similar to other *Brucella* species. Our findings reveal clear species-specific differences in *nor* operon function, providing new insights into how different *Brucella* adapt to the intracellular environments they encounter during infection.

## 2. Materials and Methods

### 2.1. Bacterial Strains and Plasmids

The bacterial strains and plasmids used in this work are listed in Table S1. All strains were stored at −80 °C in cryoprotector media: skim milk (Scharlab, Barcelona, Spain) or TYSB-7% DMSO (Tryptic Soy Broth [TSB; Scharlab, Barcelona, Spain], supplemented with 0.5% yeast extract [Condalab, Madrid, Spain], and dimethyl sulphoxide [VWR, Darmstadt, Germany]). All *Brucella* spp. were handled under BSL-3 containment in the laboratory facilities of the Universidad de Navarra, Spain (A/ES/18/I-22).

### 2.2. Bacterial Growth and Survival Conditions

Bacteria were routinely grown at 37 °C in TSB (Scharlab, Barcelona, Spain) or on TSB supplemented with European Bacteriological Agar (TSA; Condalab, Madrid, Spain). When indicated, growth media were supplemented with 20 mM NaNO_3_, 50 µg/mL kanamycin (Km; Sigma, St. Louis, MO, USA), 25 µg/mL nalidixic acid (Nal; Sigma), and/or 5% sucrose (PanReac AppliChem, ITW Reagents, Barcelona, Spain). The minimal medium used for in vitro phenotypic characterization of the mutants was that described by Gerhardt et al. [29]. To assess the effect of nitrosative stress, cultures were supplemented at inoculation with 1 mM of either nitric oxide donor MAHMA NONOate or DEA NONOate (Sigma-Aldrich, Darmstadt, Germany).

To evaluate bacterial survival, cultures of each strain were adjusted to an initial inoculum of approximately 10^9^ CFU/mL. Bacteria were then incubated in TSB supplemented with 20 mM NaNO_3_ at 37 °C under either aerobic conditions or anaerobic conditions simulated with BD GasPak EZ Anaerobe sachet systems (Becton Dickinson, Aalst, Belgium). After 7 and 14 days of incubation, cultures were serially diluted and plated on TSA, and colony-forming units (CFU) were enumerated and expressed as mean log_10_ CFU.

For growth curve analysis, bacteria were first cultured in TSB at 37 °C with orbital agitation. After 18 h, cells were harvested by centrifugation (15,700× *g*, 5 min) and resuspended in 10 mL of either TSB or Gerhardt’s medium to an optical density at 600 nm (OD_600_) of 0.1. After another 18 h of incubation with agitation at 37 °C, bacteria were harvested by centrifugation and resuspended in 1 mL of the medium at an OD600 = 0.1. Bacteria inocula were then transferred to Bioscreen plates (200 μL/well) and growth was monitored as the absorbance at 420–580 nm in a Bioscreen C (Growth Curves Ltd., Turku, Finland) every 30 min, with continuous shaking at 37 °C. Oxygen-limited conditions were simulated by overlaying cultures with sterile mineral oil (Merck, Darmstadt, Germany) to prevent oxygen diffusion. Growth curve studies were performed in triplicate and repeated at least three times.

### 2.3. DNA Manipulations

Genomic sequences of *B. abortus* 2308 were obtained from the databases National Center for Biotechnology Information (NCBI) and Kyoto Encyclopedia of Genes and Genomes (KEGG). Searches for DNA and protein homologies were carried out using NCBI BLAST (https://blast.ncbi.nlm.nih.gov/blast/Blast.cgi, accessed on 1 January 2024) [30]. Sequence alignments were performed with Clustal Omega (https://www.ebi.ac.uk/jdispatcher/msa/clustalo, accessed on 1 January 2024) [31,32]. Primers were synthesized by Condalab (Madrid, Spain). DNA sequencing was performed by Secugen (Madrid, Spain). Plasmid and chromosomal DNA were extracted with QIAprep^®^ Spin Miniprep (Qiagen, Hilden, Germany) and QIAamp^®^ DNA Mini Kit (Qiagen, Hilden, Germany), respectively. Restriction modification enzymes were used under the conditions recommended by the manufacturers.

### 2.4. Mutagenesis

For the construction of BaΔ*norD* mutant, we first generated two PCR fragments: oligonucleotides norD-F1 (5′-GCGTTGGACAAGTTGAGGTT-3′) and norD-R2 (5′-CATAGCGGTCGGTTAAATGC-3′) were used to amplify a 397 base pairs (bp) fragment including codons 1 to 11 of *norD* gene (BAB2_0365), as well as 300 bp upstream of the norD start codon. Oligonucleotides norD-F3 (5′-GTATCGCCAGCCAATTTACGTCCGTATTGGAAGCCAAGAA-3′) and norD-R4 (5′-CAGTAACAAAAGGCCGCTAT-3′) were used to amplify a 394 bp fragment including codons 606 to 633 of BAB2_0952 and 305 bp downstream of the BAB2_0952 stop codon. Both fragments were ligated by overlapping PCR using oligonucleotides F1 and R4 for amplification, and the complementary regions between R2 and F3 for overlapping. The resulting fragment, containing the *norD* deletion allele, was cloned into the *EcoR*I (Takara Bio, Kusatsu, Japan) sites of the suicide plasmid pNPTs138-Km, sequenced to ensure the maintenance of the reading frame and transformed into competent *E. coli* S17 λpir [33,34]. The resulting suicide pNPTs-derived plasmid was introduced into *B. abortus* 2308W [35,36] by double recombination. The first recombination event (integration of the suicide vector in the chromosome) was selected by Nal and Km resistance, and the second recombination (excision of the mutator plasmid leading to construction of the mutant by allelic exchange), was selected by Nal and sucrose resistance and Km sensitivity. The resulting colonies were screened by PCR with primers norD-F1 and norD-R4 which amplified a fragment of 2573 bp in the mutant and 791 bp in the sibling revertants. The absence/presence of the deleted sequence in these clones was verified using primers norD-F1 and norD-R5 (5′-GATCAAGATCGAAGCGGAAG-3′, hybridizing in the deleted region), that amplified a fragment of 1354 bp only in the wild-type strain.

### 2.5. Infection of Activated and Non-Activated RAW264.7 Macrophages

RAW264.7 macrophages (ATCC^®^ TIB-71^TM^) and THP-1 macrophage-like cells (ATCC^®^ TIB-202™) were cultured in Dulbecco’s Modified Eagle’s Medium (DMEM; Gibco, Paisley, UK) and Roswell Park Memorial Institute 1640 Medium (RPMI-1640; Gibco, Paisley, UK), respectively, both supplemented with 10% fetal bovine serum (FBS; Gibco, Paisley, UK). Cells were maintained at 37 °C in a 5% CO_2_ atmosphere for at least one week prior to infection and routinely tested negative for *Mycoplasma* contamination employing the LooKOut^®^ Mycoplasma PCR Detection Kit (Sigma-Aldrich, Darmstadt, Germany).

Infection assays were performed as described elsewhere [13]. RAW264.7 cells were seeded at 5 × 10^5^ cells/well 24 h before infection. When indicated, macrophages were stimulated for 18 h before infection with *E. coli* LPS (0.1 μg/mL) and recombinant murine IFN-γ (25 U/mL; ImmunoTools, Friesoythe, Germany). THP-1 cells were seeded at 1 × 10^5^ cells/well 48 h before infection and differentiated with 50 ng/mL phorbol 12-myristate 13-acetate (PMA; Abcam, Cambridge, UK) for 24 h pre-infection.

On infection day, cells were counted and infected at a multiplicity of infection (MOI) of 50:1. Plates were centrifuged at 400× *g* for 10 min at 4 °C and then incubated for 30 min at 37 °C in 5% CO_2_. To remove extracellular bacteria, cells were washed with fresh medium and incubated for 1 h in complete medium supplemented with 100 µg/mL of gentamicin. After that, cells were maintained in medium containing 25 µg/mL of gentamicin.

Cells were monitored daily on a light inverted microscope, and no remarkable infection-related morphological changes were observed. At 2, 24 and 48 h post-infection, cells were lysed with 0.1% Triton X-100 (Sigma-Aldrich, Darmstadt, Germany) in Dulbecco’s Phosphate-Buffered Saline (DPBS; Gibco, Paisley, UK) for 5 min at room temperature. Lysates were collected, 10-fold diluted, and plated on TSA to determine the number of intracellular bacteria.

### 2.6. Virulence Assay in Mice

Seven-week-old female BALB/c mice (Envigo, Bicester, UK) were housed in the BSL-3 facilities of CITA (ES502970012025 and A/ES/17/I-30) for 1 week before and during the experiments, with water and food ad libitum. All animal handling and experimental procedures were in accordance with the current European (Directive 2010/63/UE) and Spanish (RD 53/2013) regulations, supervised by the Ethical Committee for Animal Experimentation, and authorized by Aragón Government (reports No. 2020-03 and 2020-04).

For the virulence assay, groups of ten BALB/c mice were inoculated intraperitoneally with 10^5^ CFU of the corresponding strain in 0.1 mL of buffered saline solution (BSS; 0.015 M NaCl, 7 mM KH_2_PO_4_, 10 mM K_2_HPO_4_; pH 6.85). Inoculum doses were retrospectively assessed by plating inocula countable dilutions. The *B. abortus* 2308W strain was used as a parental control. Animal welfare was tracked daily, and no signs of illness due to inoculation with *Brucella* were found. At two- and eight-weeks post-infection, five mice per group were euthanized by cervical dislocation, and the mean CFU values per spleen were determined as reviewed elsewhere [37]. The identity of the recovered strains was confirmed by PCR from the isolates obtained from each individual mouse.

### 2.7. Statistical Analysis

Statistical comparisons for cell and mouse infection experiments were performed using unpaired t-tests. Data from in vitro survival assays were analyzed using the Mann–Whitney U test. All analyses were carried out using GraphPad Prism v9.5.1 (GraphPad Software, San Diego, CA, USA).

## 3. Results and Discussion

### 3.1. NorD Is Dispensable for Brucella abortus 2308W Growth and Survival Under Nitrate Reduction Promoting Conditions

To study the role of the *nor* operon in *B. abortus* 2308W, we generated a non-polar deletion mutant in the *norD* gene, named BaΔ*norD*, confirmed by PCR and sequencing. *norD* putatively encodes a membrane-associated component of the nitric oxide reductase complex (Nor), involved in the reduction of nitric oxide (NO) to nitrous oxide (N_2_O).

**Figure 2:**
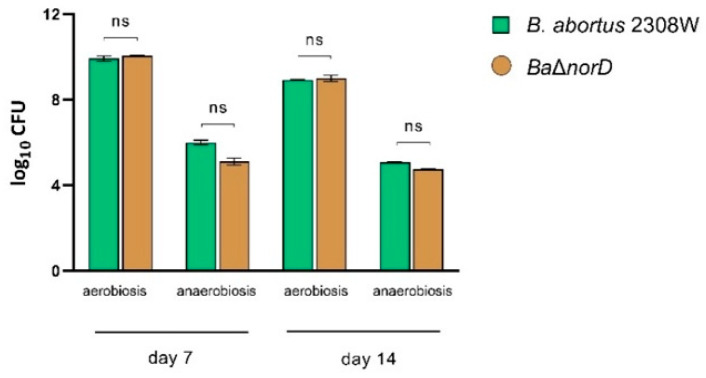
Survival of *B. abortus* 2308W and Δ*norD* under aerobic and anaerobic conditions in nitrate-supplemented medium. The experiment was repeated three times with similar results. Bars represents the mean ± standard deviation of log_10_ CFU from triplicate samples (ns = not significant).

We first assessed whether NorD contributes to long-term survival when oxygen becomes limiting. We incubated *B. abortus* 2308W wild-type and the Δ*norD* mutant for 14 days in TSB supplemented with 20 mM NaNO_3_ under either aerobic or anaerobic static conditions. Both strains behaved identically in aerobic cultures, and exhibited a similar decline in CFU under anaerobic conditions (Figure 2). These results suggest that deletion of *norD* does not impair bacterial survival under these conditions.

This finding contrasts with the phenotype described for *B. suis* 1330, in which a *norD* mutant showed marked loss of viability during anaerobic incubation in nitrate-supplemented media [25]. This phenotype was interpreted as a failure to detoxify endogenously generated NO during denitrification. The lack of a similar phenotype in *B. abortus* 2308W indicates that, under the tested conditions, this strain does not accumulate NO to levels detrimental for survival.

**Figure 3:**
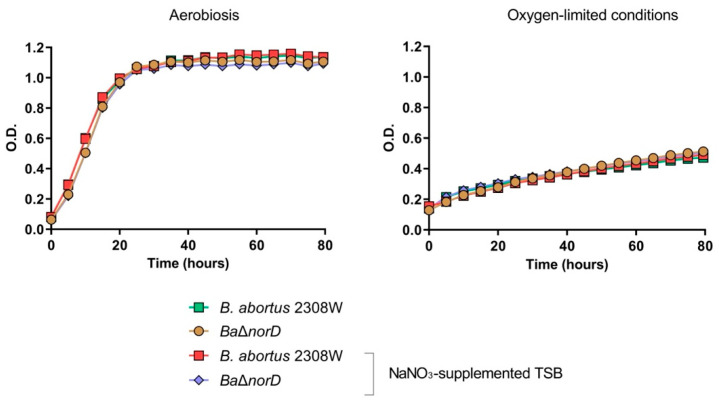
Growth curve analysis under aerobic and oxygen-limited conditions in the presence or absence of NaNO_3_. The experiment was repeated three times with similar results. Each point represents the mean standard deviation (error bars are within the size of the symbols) of optical density (OD) values of triplicate samples.

To further explore the ability of *B. abortus* 2308W to utilize nitrate, we monitored the growth of the wild-type and Δ*norD* strains in TSB, with or without NaNO_3,_ under aerobic or oxygen-limited conditions. In aerobic cultures, nitrate supplementation did not affect the growth dynamics of either strain, as measured by optical density (Figure 3), with comparable doubling times in the absence or presence of nitrate (5.5 h vs. 5.3 h for the wild-type and 4.7 h vs. 4.8 h for BaΔ*norD*, respectively). Likewise, under oxygen-limited conditions, neither strain displayed enhanced growth when nitrate was added. Instead, both strains showed doubling times between *ca.* tenfold longer than those measured aerobically, consistent with the difficulties of *B. abortus* 2308W to sustain efficient growth under these restrictive conditions.

These results indicate that nitrate supplementation does not promote growth of *B. abortus* 2308W in either oxygen-rich or oxygen-limited conditions, regardless of NorD expression. These findings are consistent with those reported by Freddi et al. [20], who reported that *B. abortus* and *B. melitensis* exhibit minimal anaerobic growth when supplemented with nitrate, in contrast to *B. suis* and especially *B. microti*, which demonstrated robust nitrate-dependent anaerobic multiplication. In fact, in *B. abortus* and *B. melitensis*, Freddi et al. detected only a slight increase in CFU counts under these conditions, while no appreciable growth was observed by optical density measurements. Our findings and those of Freddi et al. (2023) [20] suggest that *B. abortus* and *B. melitensis* exhibit limited capacity for nitrate-dependent oxygen-limited growth, likely due to an inefficient denitrification pathway.

In this sense, Rest & Robertson [24] characterized the electron transport chain of the British *B. abortus* strain 19 grown on tryptose, yeast extract, vitamins, salts, and glucose, and detected a membrane-linked nitrate reductase activity even under vigorous aeration, possibly due to oxygen depletion in dense cultures. In parallel, Sperry & Robertson [23] demonstrated that nitrate could substitute for oxygen in supporting erythritol catabolism while nitrite alone had no stimulatory effect on metabolism. These results suggested that, in *B. abortus* strain 19, the electron transport chain is coupled to a terminal nitrate reductase but not to downstream nitrite or nitric oxide reductases.

However, it is noteworthy that *B. abortus* 2308 *narK* is annotated as a pseudogene [38]. NarK functions as a dual transporter mediating nitrate uptake and nitrite extrusion [39]. Consistent with this annotation, we confirmed that *B. abortus* 2308 NarK carries a 507–amino acid deletion in the N-terminal region (Figure 1 and Table S2). Because nitrate and nitrite are charged molecules with negligible passive diffusion across membranes, this truncation might compromise nitrate import in *B. abortus* 2308, although alternative transporters could partially compensate for this defect. In addition, we observed that *narR*, which encodes the putative transcriptional activator of nitrate reductase genes, is absent in *B. abortus* 2308 (Figure 1). Notably, Haine et al. demonstrated that NarR is required for full *narK* expression in *B. melitensis* 16M [26].

Although the physiological implications of this genomic configuration are unknown and currently under study, the presence of a truncated *narK* gene and the absence of *narR* in *B. abortus* 2308 are consistent with our observation that neither nitrate supplementation nor deletion of *norD* alters survival or growth under anaerobic or oxygen-limited conditions in *B. abortus* 2308W. These genomic traits are likely to restrict nitrate import and reduce expression of nitrate reductase components, thereby limiting the activation of the denitrification pathway in this strain. Interestingly, other *B. abortus* strains, including S19, do not present the genomic defects affecting *narK* and *narR*, which may explain the differences between the nitrate reductase activity in S19 reported by Robertson et al. [23,24] and our results in *B. abortus* 2308W. Taken together, our genomic and phenotypic data indicate that *B. abortus* 2308W relies minimal, if at all, on nitrate respiration, providing a coherent explanation for why NorD is dispensable under the conditions tested.

Furthermore, comparative analyses of the proteins encoded by the *nar*, *nos*, *nir* and *nor* operons across *Brucella* species revealed multiple amino acid substitutions and differences in the annotated start sites of several genes (Table S2). Although the overall organization of these operons is conserved (Figure 1), such sequence-level divergence is likely to differentially affect protein function across species. Overall, while the operons retain their gene organization (Figure 1), these sequence-level modifications may affect the function of the system differently among *Brucella* species. The functional consequences of these genomic variations, particularly in terms of enzyme activity and pathway efficiency, are currently under investigation.

### 3.2. Deletion of NorD Does Not Affect Resistance of Brucella abortus 2308W to Nitrosative Stress

Nitrosative stress is one of the main immunological barriers faced by intracellular pathogens during infection. Although we did not observe evidence of nitrate reduction in *B. abortus* 2308W, the *nor* operon could still function in detoxification of exogenous NO, as proposed for other *Brucella* species, including *B. suis* and *B. melitensis* [25,26].

**Figure 4:**
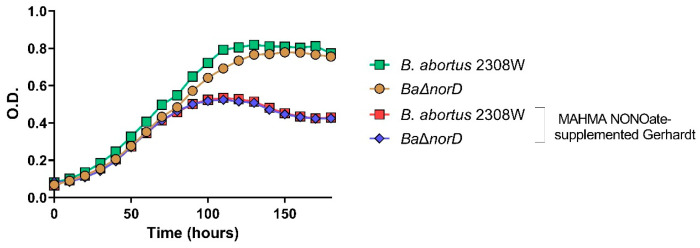
Effect of the NO donor MAHMA NONOate on the growth of *B. abortus* 2308W and Δ*norD*. The experiment was repeated three times with similar results. Each point represents the mean and standard deviation (error bars are within the size of the symbols) of optical density (OD) values of triplicate samples.

To evaluate this, we compared the growth of *B. abortus* 2308W wild-type and the ∆*norD* mutant in Gerhardt’s minimal medium supplemented with nitric oxide-releasing compounds: MAHMA NONOate and DEA NONOate (Figure 4). In the absence of nitrosative stress, both strains displayed comparable growth rates with no significant differences. As expected, exposure to either NO donor led to a marked growth reduction in both strains, confirming the effective induction of nitrosative stress. However, the degree of growth inhibition was similar for the wild-type and the ∆*norD* mutant, indicating that *norD* deletion does not increase susceptibility to NO under these in vitro conditions.

These results contrast with those reported for *B. melitensis* 16M, where deletion of *norB*, increased susceptibility to NO [26]. This discrepancy suggests that the role of NorD in nitrosative stress resistance might be species-dependent. In *B. abortus* 2308W, the absence of a phenotype may reflect low *nor* operon expression under the tested conditions or the presence of compensatory NO-detoxifying systems, as reported in other bacteria [40]. In line with our results, a recent study showed that quorum sensing regulates the denitrification pathway in *B. abortus* 2308 but deletion of quorum-sensing components did not affect NO detoxification or growth under oxygen limited denitrifying conditions [41].

### 3.3. NorD Is Not Required for B. abortus 2308W Survival or Replication in Macrophages or in the Mouse Model

During infection, host immune cells produce NO through inducible NO synthase (iNOS), generating nitrosative stress conditions with dynamics and concentrations that differ from those generated by NO donors in growth medium. Although no differences in NO susceptibility between the wild-type and mutant strain were observed under in vitro conditions, we next examined whether NorD may be required under more physiological, host-mimicking conditions.

To address this question, we first stimulated RAW264.7 murine macrophages with *E. coli* LPS and IFN-γ, and then infected them with *B. abortus* 2308W wild-type or the Δ*norD* mutant. Parallel infections were performed in non-stimulated macrophages. NO production in activated cells was confirmed using the Griess reagent, whereas nitrite levels were undetectable in supernatants from non-stimulated cells.

**Figure 5:**
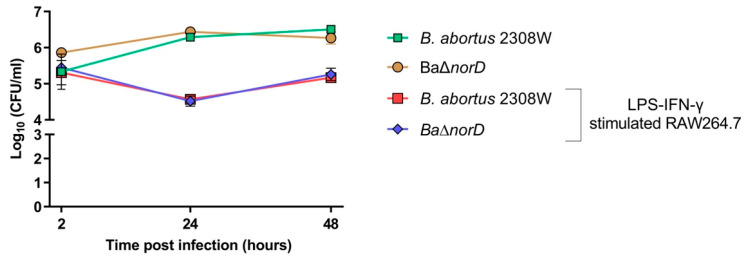
Intracellular multiplication in RAW264.7 macrophages stimulated with *E. coli* LPS and IFN-γ and not stimulated. The experiment was repeated three times with similar results. Each point represents the mean ± standard deviation of triplicate wells from one representative experiment.

As shown in Figure 5, in activated RAW264.7 macrophages, both *B. abortus* 2308W wild-type and ∆*norD* exhibited a significant reduction in CFU counts between 2 and 24 h post-infection, consistent with effective killing by the cells. Bacterial loads remained low at 48 h, and no significant differences were observed between the two strains at any time point, indicating that NorD is not required for survival or replication in activated macrophages under nitrosative stress conditions. In non-stimulated macrophages, both strains replicated progressively over the 48 h period, with increasing CFU counts and no significant differences between them (Figure 5). The absence of an early decline in CFU levels in these conditions confirms the lack of strong antimicrobial activity in resting macrophages and supports that the LPS/IFN-γ stimulation protocol effectively triggered a functional response. Similar results were obtained in non-stimulated THP-1 cells (Figure S1). Taken together, these data indicate that NorD is dispensable for resistance to physiological nitrosative stress in this macrophage infection model.

**Figure 6:**
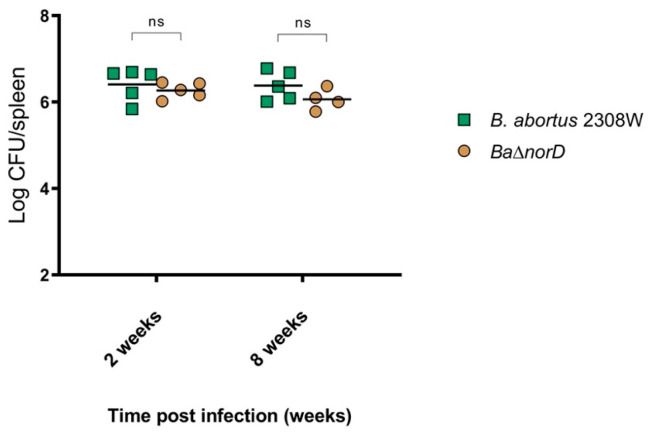
Bacterial loads in the spleens of BALB/c mice at 2 and 8 weeks post-infection. Bacterial burden at the spleens was determined by CFU counting and is expressed as mean log10 CFU/spleen ± standard deviation (ns = not significant).

To evaluate whether NorD contributes to virulence in vivo, BALB/c mice were infected intraperitoneally with 10^5^ CFU of either *B. abortus* 2308W or the Δ*norD* mutant, and splenic colonization was measured at 2 and 8 weeks post-infection. At both time points, bacterial loads in the spleen were comparable between the two strains, with no statistically significant differences (Figure 6), indicating that *norD* deletion does not compromise the ability of *B. abortus* 2308W to persist and replicate in the murine model.

Together, these results demonstrate that NorD is dispensable for *B. abortus* 2308W intracellular survival in both resting and activated macrophages, as well as for multiplication in the mouse spleens. This finding contrasts with studies in other *Brucella* species. In *B. suis* 1330, Loisel-Meyer et al. reported a strong attenuation of a Δ*norD* mutant in activated macrophages, but not in resting cells, and in mice, indicating a key a role for Nor-mediated resistance to host-derived NO [25]. In *B. melitensis* 16M, Haine et al. showed that deletion of *norB* (and of its regulator *nnrA*) similarly impaired survival in activated J774 macrophages and reduced persistence in mice [26]. Importantly, this attenuation was reversed when macrophages were treated with the NO synthase inhibitor L-NAME, directly linking the phenotype to host-derived NO. These findings, together with the fact that attenuation occurs only in activated cells, suggest that *B. melitensis* relies on detoxification of host-produced NO, a notion consistent with the analysis by Wang et al. showing that *B. abortus* S2308 can adapt to a high-NO environment by expressing genes that either neutralize its toxic effects or allow the bacterium to exploit NO as a nitrogen source [42]. Moreover, Wang et al. reported that *B. abortus* S2308 inhibits macrophage apoptosis under nitrosative stress, a mechanism that likely promotes bacterial persistence within host cells. Lestrate et al. found that mutation of *norE* in *B. melitensis* 16M reduced the ability of *B. melitensis* 16M to invade and persist within non-activated bovine macrophages and HeLa cells, and in mice [43]. This alternative scenario supports the view, as Wang et al. had suggested, that *Brucella* may replicate in oxygen-deprived niches by switching to anaerobic growth with nitrogen compounds as electron acceptors [42]. Compatible with both hypotheses, Rossetti et al. reported up-regulation of *B. melitensis* 16M *narG*, *norB*, and *nosZ* during HeLa cells infection [44]. Supporting the role of nitrate respiration, Kohler et al. and Kim et al. independently showed that *narG* mutants are attenuated: Kohler et al. reported attenuation of a *B. suis* 1330 *narG* mutant in THP-1 macrophages [45], while Kim et al. observed reduced intracellular replication of *B. abortus* 544 *narG* mutants in HeLa and RAW 264.7 cells [46]. Nevertheless, because both mutants were generated by transposon mutagenesis, polar effects on downstream operons cannot be excluded. Consistent with the role of denitrification genes in shaping virulence, Baek et al. showed that complementation of the *nirK*–*nirV*–*nnrA* region in *B. neotomae* reduced virulence in IRF-1^−/−^ mice [47]. This paradoxical attenuation may result from increased intracellular NO production and suggests that the loss of these genes might represent an adaptive strategy in *B. neotomae*.

Overall, and integrating all the results presented in this study, our observations highlight a major difference among *Brucella* spp., while NorD disruption severely compromises virulence in *B. suis* and, to a lesser extent, mutations in related genes impair survival in *B. melitensis*, NorD appears dispensable in *B. abortus 2308*. It is tempting to speculate that the discrepancies might reflect differences in nitrate respiration capacity among *Brucella*. *B. suis* exhibits a higher ability to respire nitrate anaerobically, and disruption of NorD severely impairs virulence. In contrast, *B. abortus* 2308 shows little or no capacity to grow under anaerobic nitrate-respiring conditions, which is in line with the genome defect in *nark* and *narR* and may explain why Δ*norD* mutants remain fully virulent in this strain. In *B. melitensis*, attenuation of a Δ*norB* mutant was comparatively mild, which could also be related to its lower nitrate respiration capacity compared to *B. suis*, as described by Freddi et al. [20]. Thus, the contribution of NorD to virulence may parallel the extent to which each species depends on nitrate respiration as an adaptive strategy during infection. At the same time, the role of the *nor* operon in NO detoxification cannot be overlooked. This dual function suggests that variation in both nitrate respiration and NO detoxification capacities converge in vivo to determine the impact of NorD on pathogenesis. Finally, although the *nor* operon may participate in additional cellular processes not covered in this study, our results indicate that such potential roles are not essential for *B. abortus* 2308W virulence under the conditions evaluated.

Taken together, these results reveal functional divergence within the *nor* operon across classical *Brucella* species and highlight the need for further studies to elucidate how nitrogen metabolism and bacterial defenses against host-derived NO intersect to shape virulence, likely reflecting evolutionary strategies in which species that do not elicit strong host NO responses rely less on Nor-mediated functions and therefore exhibit limited functional conservation of this pathway.

## 4. Conclusions

Our results demonstrate that the deletion of *norD* in *B. abortus* 2308W does not affect bacterial growth, resistance to nitrosative stress, intracellular survival, or virulence in mice. This contrasts with previous reports in *B. suis* and *B. melitensis*, where mutations in genes of the *nor* operon resulted in significant attenuation in vitro or in vivo. These findings indicate that the role of NorD is species- and strain-dependent and suggest that *B. abortus* 2308W relies less on this component than other *Brucella* species. The divergent outcomes highlight that the relationships between denitrification genes, nitrogen metabolism, and virulence are complex, and underline the need for further research to unravel how *Brucella* species differentially exploit nitrogen-related pathways during infection.

## Data Availability

The original contributions presented in this study are included in the article/Supplementary Materials. Further inquiries can be directed to the corresponding authors.

## Supplementary Materials

The following supporting information can be downloaded at: https://www.mdpi.com/article/10.3390/microorganisms13122875/s1, Table S1: Bacterial strains and plasmids used in this work. Table S2: Analysis of amino acid sequences in the *Brucella* species: *B. abortus* 2308, *B. melitensis* ATCC 23457, *B. melitensis* bv. 1 16M, *B. microti*, *B. suis* 1330, *B. canis* ATCC 23365, *B. suis* ATCC 23445 and *B. ovis* ATCC 25840. Figure S1: Intracellular multiplication in THP-1 derived macrophages. *B. abortus* 2308W and Δ*norD* showed similar intracellular survival.

## Abbreviations

The following abbreviations are used in this manuscript:

ATCC	American Type Culture Collection
BSS	Buffered Saline Solution
BSL3	Biosafety Level 3
CFU	Colony Forming Unit
CO_2_	Carbon Dioxide
DMEM	Dulbecco’s Modified Eagle Medium
DMSO	Dimethyl Sulfoxide
DNA	Deoxyribonucleic Acid
DPBS	Dulbecco’s Phosphate-Buffered Saline
FBS	Fetal Bovine Serum
iNOS	Inducible Nitric Oxide Synthase
KEGG	Kyoto Encyclopedia of Genes and Genomes
Km	Kanamycin
LPS	Lipopolysaccharide
MOI	Multiplicity of Infection
NaNO_3_	Sodium Nitrate
Nal	Nalidixic Acid
N_2_	Dinitrogen Gas
N_2_O	Nitrous Oxide
NCBI	National Center for Biotechnology Information
NO	Nitric Oxide
NO_2_^−^	Nitrite
NO_3_^−^	Nitrate
OD600	Optical Density at 600 nm
PCR	Polymerase Chain Reaction
SD	Standard Deviation
TSA	Tryptic Soy Agar
TSB	Tryptic Soy Broth
