# Supplementary material for: Disruption of the Nitric Oxide Reductase Operon via norD Deletion Does Not Affect Brucella abortus 2308W Virulence
Source: Microorganisms. 2025 Dec 18;13(12):2875. doi: 10.3390/microorganisms13122875 (PMC12736344; doi:10.3390/microorganisms13122875)
Supplement: Supplementary file 1 [file microorganisms-13-02875-s001.zip › microorganisms-3963557-supplementary.pdf]

Table S1. Bacterial strains and plasmids used in this work.

Strain / Plasmid	Characteristics	Reference
***Brucella* spp.**		
B. abortus	*Brucella abortus* 2308 virulent biovar 1, smooth LPS, spontaneous nalidixic acid resistant.	[33,34]
BaΔ*norD*	*B. abortus* carrying an internal deletion in *norD* ($\Delta_{12\text{-}605}$)	This work
Escherichia coli		
Stellar	F–, endA1, supE44, thi-1, recA1, relA1, gyrA96 phoA, Φ80d lacZΔ M15, Δ(lacZYA-argF) U169 Δ(mrr-hsdRMS-mcrBC), ΔmcrA, λ–	Clontech
S17- λpir	TpR StrpR recA thi hsdRM+, lambda pyr phage lysogen RP4::2-Tc::Mu::Km Tn7	[31,32]
**Plasmids**		
pNPTs138	Suicide vector; Km^R; Sac^S	[12]
pNPTsΔ*norD*	pNPTs suicide plasmid containing the *norD* deleted allele	This work

Table S2: analyzed: KEGG sequences of *B. abortus* 2308, *B. melitensis* ATCC 23457, *B. melitensis* bv. 1 16M, *B. microti*, *B. suis* 1330, *B. canis* ATCC 23365, *B. suis* ATCC 23445, *B. ovis* ATCC 25840.

**Gene**	**Gene ID (*B. abortus*)**	**Aminoacid and sequence variations**
narB / narR	-	Absent in *B. abortus.*
narK	BAB2_0902 (*pseudogen*)	Start codon in *B. abortus* 2308 annotated 1572 nucleotides after the one in the rest of the analyzed species.<br>Start codon in *B. melitensis* 16M annotated 51 nucleotides before the rest of species, but sequences coincide.<br>3 (T→A in *B. melitensis* 16M and ATCC)<br>39 (G→S in *B. melitensis* 16M)<br>56 (A→V in *B. melitensis* 16M)<br>166 (Y→F in *B. melitensis* 16M and ATCC)<br>683 (T→I in *B. suis* 1330)<br>773 (A→V in *B. melitensis* ATCC)<br>780 (A→T in *B. canis*)<br>796 (M→V in *B. canis*)<br>From aminoacid 881 to the end of the protein, *B. abortus* 2308, *B. melitensis* 16 and *B. melitensis* ATCC are different from the rest of species and have 39 aminoacids more.
narG	BAB2_0904	Start codon in *B. melitensis* 16M annotated 336 aminoacids after the one in the rest of the analyzed species (may be incorrectly annotated since the gene sequence coincides).<br>Stop in *B. abortus* 2308 annotated 10 aminoacids before the rest of the analyzed species (premature stop codon).<br>175 (A→T in *B. abortus*)<br>275 (G→D in *B. canis* and *B. suis* 1330)<br>456 (R→S in *B. abortus* 2308)<br>1229 (K→T in *B. suis* 1330)
narH	BAB2_0905	342 (L→F in *B. suis* ATCC 23445)<br>359 (L→F in *B. melitensis* 16M)<br>420 (E→K in *B. suis* and *B. canis*)<br>480 (V→D in *B. microti* and *B. melitensis*)
narJ	BAB2_0906	Start codon in *B. abortus* 2308 annotated 118 nucleotides after the one in the rest of the analyzed species (may be incorrectly annotated since the gene sequence coindices).

		59 (A→V in *B. suis* ATCC)<br>208 (G→S in *B. abortus* 2308)
narI	BAB2_0907	185 (V→M in *B. suis* 1330)
nnrR/nnrB	BAB2_0922	Start anotated 86 aminoacids after the rest of species in *B. melitensis* 16M.<br>33 (P→Q in *B. abortus* 2308)<br>110 (P→S in *B. suis* 1330 and *B. canis*)<br>154 (R→H in *B. suis* 1330, *B. suis* ATCC and *B. canis*)<br>166 (A→T in *B. suis* 1330 and *B. canis*)
nosX	BAB2_0923	53 (G→D in *B. abortus* and *B. microti*)<br>60-64 (RLVGES→V----K in *B. abortus*)<br>First 59 aminoacids in *B. melitensis* 16M are different.
nosL	BAB2_0924	48 (H→Y in *B. microti*)<br>118 (E→K in *B. melitensis* 16M)<br>150 (I→V in *B. ovis*)
nosY	BAB2_0925	88 (M→L in *B. abortus* 2308)<br>160 (I→T in *B. ovis*)<br>194-196 (LAL→ --- in *B. melitensis* 16M)<br>270 (V→G in *B. suis* 1330)<br>271-272 (FS→LL in *B. suis* 1330 and ATCC)<br>272 (S→L in *B. canis*)
nosF	BAB2_0926	
nosD	BAB2_0927	
nosZ	BAB2_0928	
nosR	BAB2_0929	
nnrA	BAB2_0941	23 (L→V in *B. canis*)<br>33 (R→W in *B. canis*)<br>43 (E→K in *B. suis* ATCC)<br>63 (Q→K in *B. abortus*)<br>127 (A→V in *B. abortus*)<br>149 (E→G in *B. canis*)<br>188 (T→S in *B. suis* ATCC; T→V in *B. canis*)
nirV	BAB2_0942	Start anotated 23 aminoacids after the rest of species in *B. melitensis* 16M<br>116 (H→I in *B. melitensis* ATCC and 16M)<br>148 (S→W in *B. canis*)

		157 (Q→R in *B. canis* and *B. suis* 1330)
nirK	BAB2_0943	129 (M→V in *B. suis* 1330)<br>189 (G→E in *B. canis)*<br>300 (A/K/S/F in different strains)<br>360 (V→A in *B. abortus*, *B. melitensis* and *B. microti*)
norD	BAB2_0952	73 (Q→R in *B. suis* 1330)<br>74 (R→Q in *B. microti*)<br>296 (D→G in *B. melitensis*)<br>334 (A→V in *B. melitensis* ATCC)<br>359 (D→N in *B. melitensis* 16M)<br>391 (Q→R in *B. microti*)<br>575 (S→C in *B. melitensis* ATCC)<br>582 (A→V in *B. abortus* and *B. melitensis*)<br>601 (Y→H in *B. suis* 1330)<br>624 (L→M in *B. abortus*)
norQ	BAB2_0953	56 (A→V in *B. suis* 1330)<br>122 (H→Y in *B. suis* 1330)<br>142 (A→T in *B. suis* ATCC)
norB	BAB2_0954	150-152 (QHS→TNI in *B. abortus*, *B. melitensis* 16M, *B. melitensis* ATCC and *B. microti*)<br>280 (K→E in *B. abortus*)<br>342 (V→A in *B. abortus*)<br>354 (A→T in *B. abortus*)
norC	BAB2_0955	87 (G→A in *B. suis* ATCC, *B. suis* 1330 and *B. canis*)
norF	BAB2_0956	74 (P→S in *B. suis* ATCC)
norE	BAB2_0957	Start anotated 50 aminoacids before the rest of species in *B. abortus*<br>42 (V→D in *B. melitensis* ATCC and 16M)<br>86 to 94 (RRRNNPKAV missing in *B. abortus*)<br>125 (D→N in *B. abortus*)

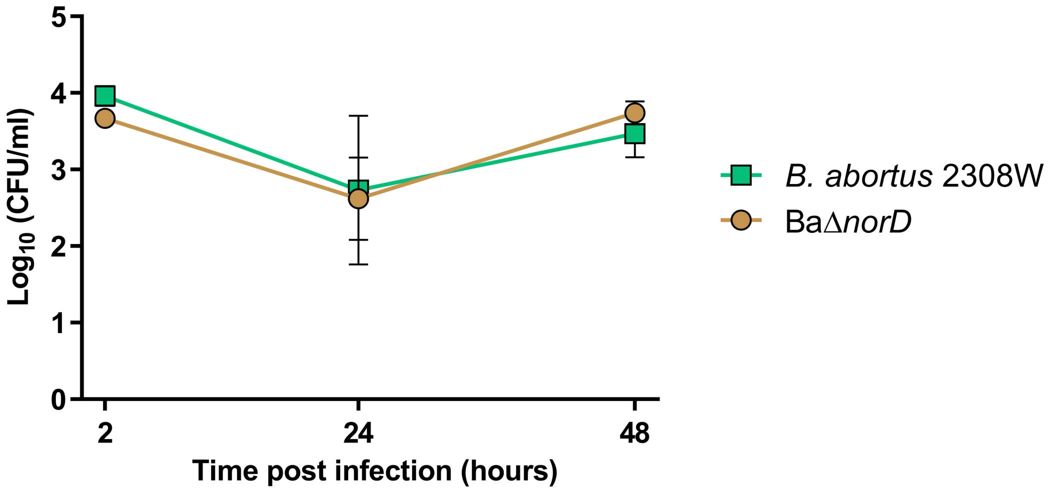

Figure S1. Intracellular multiplication in THP-1 derived macrophages. *B. abortus* 2308W and Δ*norD* showed similar intracellular survival.
